# Cross-cultural adaptation and reliability and validity of the Dutch Patient-Rated Tennis Elbow Evaluation (PRTEE-D)

**DOI:** 10.1186/1471-2474-15-270

**Published:** 2014-08-11

**Authors:** Mathijs van Ark, Johannes Zwerver, Ronald L Diercks, Inge van den Akker-Scheek

**Affiliations:** University of Groningen, University Medical Center Groningen, Center for Sports Medicine, University Center for Sport, Exercise and Health, P.O. Box 30.001, 9700 RB Groningen, The Netherlands

**Keywords:** Arm injuries, Tennis elbow, Tendon, Tendinopathy, Lateral epicondylitis, PRTEE, PRFEQ

## Abstract

**Background:**

Lateral Epicondylalgia (LE) is a common injury for which no reliable and valid measure exists to determine severity in the Dutch language. The Patient-Rated Tennis Elbow Evaluation (PRTEE) is the first questionnaire specifically designed for LE but in English. The aim of this study was to translate into Dutch and cross-culturally adapt the PRTEE and determine reliability and validity of the PRTEE-D (Dutch version).

**Methods:**

The PRTEE was cross-culturally adapted according to international guidelines. Participants (n = 122) were asked to fill out the PRTEE-D twice with a one week interval to assess test-retest reliability. Internal consistency of the PRTEE-D was determined by calculating Crohnbach’s alphas for the questionnaire and subscales. Intraclass Correlation Coefficients (ICC) were calculated for the overall PRTEE-D score, pain and function subscale and individual questions to determine test-retest reliability. Additionally, the Disabilities for the Arm, Shoulder and Hand questionnaire (DASH) and Visual Analogue Scale (VAS) pain scores were obtained from 30 patients to assess construct validity; Spearman’s correlation coefficients were calculated between the PRTEE-D (subscales) and DASH and VAS-pain scores.

**Results:**

The PRTEE was successfully cross-culturally adapted into Dutch (PRTEE-D). Crohnbach’s alpha for the first assessment of the PRTEE-D was 0.98; Crohnbach’s alpha was 0.93 for the pain subscale and 0.97 for the function subscale*.* ICC for the PRTEE-D was 0.98; subscales also showed excellent ICC values (pain scale 0.97 and function scale 0.97). A significant moderate correlation exists between PRTEE-D and DASH (0.65) and PRTEE-D and VAS pain (0.68).

**Conclusion:**

The PRTEE was successfully cross-culturally adapted and this study showed that the PRTEE-D is reliable and valid to obtain an indication of severity of LE. An easy-to-use instrument for practitioners is now available and this facilitates comparing Dutch and international research data.

**Electronic supplementary material:**

The online version of this article (doi:10.1186/1471-2474-15-270) contains supplementary material, which is available to authorized users.

## Background

Lateral Epicondylalgia (LE), also known as tennis elbow, is a common injury with a high prevalence especially in a 40–50 year old population [1, 2]. The prevalence in the general population (25–64 years) is 1.3% for males and 1.1% for females [3].

LE is in most cases a tendinopathy of the Extensor Carpi Radialis Brevis tendon caused by overuse of the extensor tendons of the forearm [4]. It is characterized by pain and tenderness near the lateral epicondyle of the humerus, a weak and painful grasp and pain with extension of the wrist and the third metacarpal [5]. Despite the prevalence of LE, only little consensus exists on its treatment [6, 7]. Examples of treatments used for LE in practice are (eccentric) exercise programs, acupuncture, injections, taping, ESWT and deep friction massage. Further randomized and controlled studies with reliable outcome measures including questionnaires are required to determine the optimal treatment strategy.

The first questionnaire specifically designed for LE was developed in Canada in 1999. This questionnaire was called the Patient-Rated Forearm Evaluation Questionnaire (PRFEQ) [8]. The PRFEQ was developed to provide a brief, uncomplicated, standardized quantitative description of pain and functional ability for use in patients with LE to assess severity [8]. The PRFEQ is found to be reliable and sensitive [8, 9]. In 2005, some minor changes were made in the wording of the PRFEQ along with a change of the name in PRTEE (Patient-Rated Tennis Elbow Evaluation) to improve the questionnaire [10]. The developers state that the published reliability and validity data still apply, because only minor changes were made to the PRFEQ.

The English-language PRTEE has already been translated and cross-culturally adapted in Italian, Swedish, Turkish and Canadian-French [11–14]. Previously the PRFEQ was translated and cross-culturally adapted into Hong Kong Chinese [15]. In the Dutch language, less specific questionnaires for the upper extremity, like the Disabilities for the Arm, Shoulder and Hand (DASH) questionnaire, exist. However, a reliable and valid questionnaire specific for measuring patient perceived severity of LE is not yet available in Dutch. The cross-cultural adaptation of the PRTEE would provide such a questionnaire and this would be another step for a universally accepted outcome measure for LE.

Therefore, the aim of this study is to translate into Dutch and cross-culturally adapt the PRTEE according to international guidelines [16]. Furthermore, the reliability and validity of the Dutch version of the PRTEE will be determined.

## Methods

### Study design

The PRTEE was cross-culturally adapted to the Dutch language. Subsequently internal consistency, test-retest reliability and construct validity were assessed. The Medical Ethical committee of the University Medical Center Groningen reviewed the study protocol and concluded that the study was not subject to the Medical Research Involving Human Subjects Act. No formal ethical approval was therefore needed. All participants received the PRTEE-D questionnaire with an accompanying letter, informing about the study and its goals and explaining that return of the questionnaire will be taken as consent to participate.

### Cross-cultural adaptation

Permission for the cross-cultural adaptation of the PRTEE to Dutch was obtained from the developer of the PRTEE (personal communication, Dr. J.C. MacDermid). The cross-cultural adaptation was performed according to the five stage guideline for this process in self-report measures [16].

### Stage 1: forward translation

The English PRTEE was translated into Dutch by two translators. One translator had a medical background and was aware of the purpose of the translation. The other translator did not have a medical background and was not aware of the purpose of the translation.

### Stage 2: synthesis of the translations

A synthesis of both translations was developed by reaching consensus between the two translators and an observer. This synthesis process was documented in a written report.

### Stage 3: back translation (to English)

Two bilingual native English speakers translated the synthesized version of stage 2 back to English. They were not familiar with the research protocol, the concepts explored or the PRTEE.

### Stage 4: expert committee

An expert committee consisting of a sports medicine physician, human movement scientist, epidemiologist and the translators reached consensus on a translation of the PRTEE. All previous translations of the PRTEE were taken into consideration to reach this consensus. The expert committee meeting resulted in a pre-final version of the PRTEE-D (Patient-Rated Tennis Elbow Evaluation – Dutch).

### Stage 5: pretesting

The final stage of the cross-cultural adaptation of the PRTEE was pretesting of the questionnaire. Ten persons filled out the PRTEE-D. After completing the questionnaire each subject was asked to point out any difficulties in understanding or ambiguities in the questionnaire.

### Reliability

‘Reliability’ is a generic term used to indicate both the homogeneity (internal consistency) of a scale and the reproducibility (test–retest reliability) of scores [17]. Both were determined for the PRTEE-D. The PRTEE-D was filled out by 90 healthy participants recruited at universities and tennis clubs and 32 LE patients diagnosed by a physical therapist or sports medicine physician. Physiotherapists, general practitioners and sports physicians in and in the area of the University Medical Center Groningen were contacted. The clinicians asked patients with diagnosed LE to participate in the study. Patients were asked to complete the questionnaire twice with an interval of 1 week to assess test-retest reliability [17].

### Validity

To assess construct validity, the patients also filled out the DASH questionnaire and indicated degree of pain in their arm on a Visual Analogue Scale (VAS) the first time they filled out the PRTEE-D. Criterion validity of the PRTEE-D was not assessed, because a ‘gold standard’ or other questionnaires measuring severity of LE do not exist.

### Questionnaires

#### PRTEE

The PRTEE provides a score of pain and functional ability of LE patients over the last week. The questionnaire consists of two subscales: a pain scale and a function scale; five questions regarding pain in the elbow and ten questions regarding the function of the elbow. Answers have to be given on an eleven point scale with 0 representing no pain or difficulty in performing a task and 10 representing the worst pain imaginable or unable to do the task. The maximum score is 50 for the pain subscale and 100 for the function subscale. The function subscale was divided by 2. The total score was calculated by adding the scores of the pain and function subscales.

#### DASH

The Dutch language version of the DASH questionnaire was found to be reliable and valid for assessing disability and symptoms in patients with upper limb disorders [18]. It was designed for any condition in the upper limb [19]. The DASH questionnaire consists of 30 items, 21 are physical function items, 6 symptom items and 3 social or role function items. Items refer to situations in the last week. Answers had to be given on a 5-point Likert scale, ranging from no difficulty to unable, from none to extreme, or from no impact to high impact. The raw score was transformed to a score ranging from 0 to 100. A score of 0 indicates minimal disability and 100 indicates maximal disability.

### Visual Analogue Scale (VAS) pain

Patients were asked to indicate the degree of pain in their arm by drawing a line on a scale (0-100 mm) from no pain to unbearable pain.

### Data analyses

Descriptive statistics (mean, SD) were used to describe subject’s characteristics. Test-retest reliability was determined with the Intraclass Correlation Coefficient (ICC(2,1)). This was done for the overall PRTEE-D score, pain and function subscore and scores on individual questions (Two-way mixed effect model absolute agreement). An ICC value < 0.4 was considered to be ‘poor’, a value of 0.4 – 0.75 was considered to be ‘fair to good’ and an ICC > 0.75 was considered to be excellent [20]. Additionally, to determine absolute agreement, a Bland and Altman plot was made; the mean difference (d) between the first and second assessment with corresponding 95% CI and the 95% Limits of Agreement (LOA) were displayed [21]. As proposed by Bland and Altman, an absolute agreement exists when zero lies within the 95% CI of the mean difference between test and retest measurement [21]. Internal consistency of the PRTEE-D was assessed by calculating Cronbach’s alphas for the total score and subscores. Internal consistency was considered excellent when Cronbach’s alpha exceeds 0.80, adequate when Cronbach’s alpha is between 0.70 and 0.79, and inadequate when it is lower than 0.70 [22]. Separate reliability analyses for the LE patients only were also performed as well as a factor analysis with Principal Component Analysis.

Construct validity was determined by calculating Spearman’s correlation coefficient between the PRTEE-D (subscores) and DASH and VAS-pain scores. Spearman’s rho correlations were interpreted according to Domholdt [23]: little, if any 0.00–0.25, low 0.26–0.49, moderate 0.50–0.69, high 0.70–0.89 and very high 0.90–1.00*.* An alpha < 0.05 was considered to be significant. Furthermore, a Bland Altman plot was made to determine whether systematic bias occurred between PRTEE-D and DASH questionnaire*.* All statistical tests were performed using SPSS 18.0 for Windows.

## Results

### Cross-cultural adaptation

The PRTEE was successfully cross-culturally adapted into Dutch. Back translation corresponded well with the original questionnaire, only minor differences were encountered. All members of the expert committee agreed on the pre-final version of the PRTEE-D. Pre-testing revealed that there were no difficulties in understanding or ambiguities in the PRTEE-D. Some patients indicated that they were not able to provide a good answer to some questions because they always performed that activity with their non-injured arm. The PRTEE-D is available as a supplement to this article (Additional file 1).

### Subjects

Table 1 shows the subject’s characteristics. The PRTEE-D was filled out twice by 122 participants (47 males, 75 females). Additionally, 30 LE patients (14 males, 16 females) completed the DASH questionnaire and a VAS-pain score.

**Table 1:** **Subject characteristics and descriptive statistics of the PRTEE-D, DASH and VAS pain scores**

Measure	Participants test-retest reliability n = 122 (mean, SD)	Participants construct validity n = 30 (mean, SD)
Age (years)	28.8 (13.5)	45.6 (8.9)
Height (cm)	175.1 (9.1)	176.1 (11.8)
Weight (kg)	73.0 (15.4)	89.9 (16.5)
Duration of symptoms (months)	6.6 (25.6)	20.0 (44.1)
Hours sports per week	3.9 (3.0)	3.2 (1.4)
PRTEE-D^a^	14.8 (24.1)	51.5 (18.3)
PRTEE-D pain subscore^a^	8.1 (12.7)	26.9 (8.6)
PRTEE-D function subscore^a^	6.7 (11.8)	24.6 (10.8)
DASH score		36.7 (18.9)
VAS pain score		56.8 (21.8)
% LE patients	26%	100%

^a^The first PRTEE-D measurement is used to calculate the mean score. PRTEE-D = Patient Rated Tennis Elbow Evaluation-Dutch, DASH = Disabilities for the Arm, Shoulder and Hand questionnaire, VAS = Visual Analogue Scale, LE = Lateral Epicondylalgia, SD = Standard Deviation.

### Internal consistency

The Crohnbach’s alpha for the first assessment of the PRTEE-D was 0.98. The pain subscale showed a Cronbachs Alpha of 0.93 and the function subscale 0.97. Analysis of the internal consistency for the LE patients alone showed a Crohnbach’s alpha of 0.93. The pain subscale showed a Crohnbach’s alpha of 0.80 and the function subscale 0.91 in the analysis of the LE patients. A factor analysis revealed one factor (eigenvalue = 12.04) with an explained variance of 80.3%.

### Test-retest reliability

**Figure 1:**
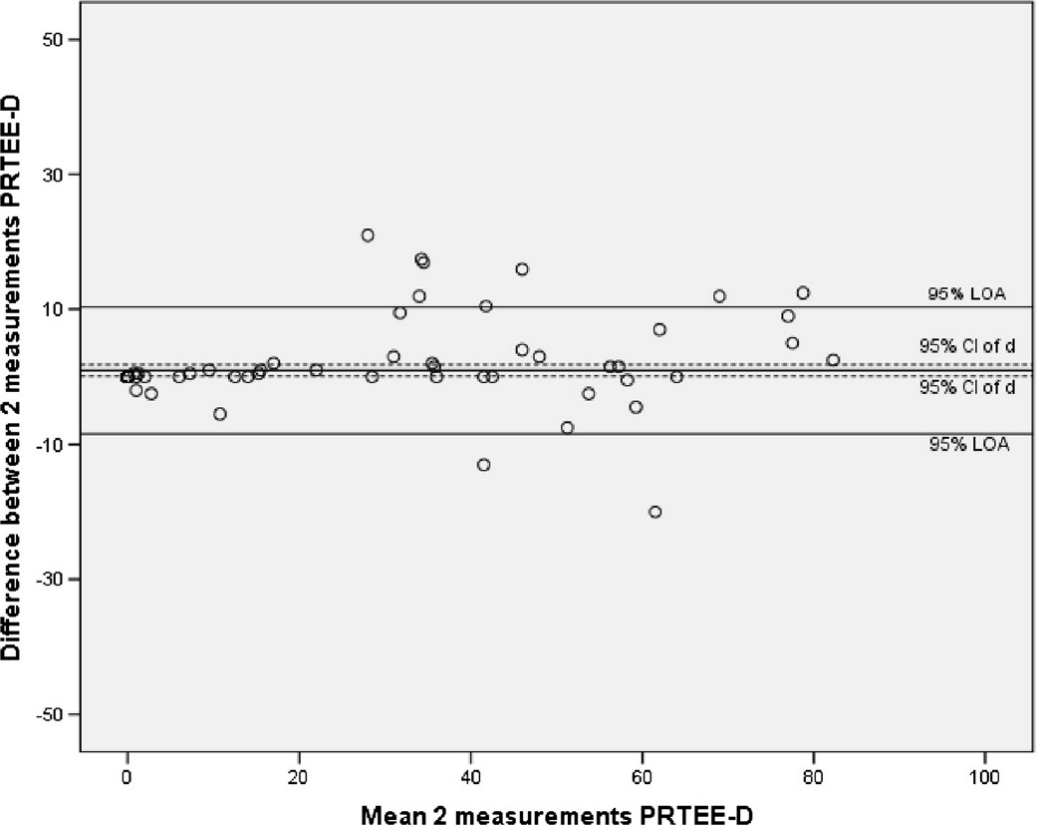
**Bland and Altman plot of reliability (agreement) of the PRTEE-D (2 measures).**

**Figure 2:**
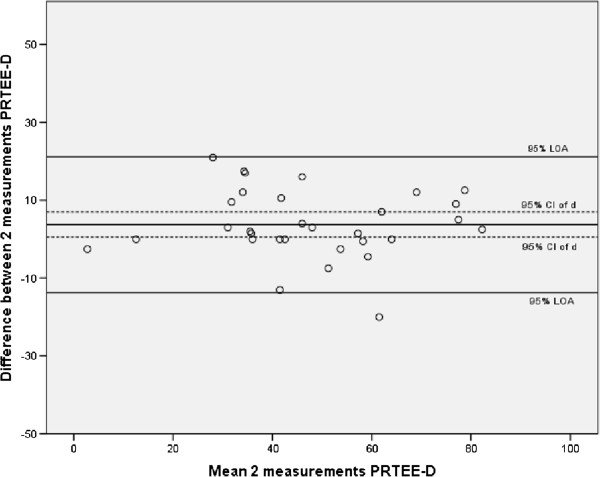
**Bland and Altman plot of reliability (agreement) of the PRTEE-D (2 measures) for LE patients.**

Table 2 shows the ICCs of the total score of PRTEE-D, subscales and individual items. ICC (2,1) values showed excellent test-retest reliability for the PRTEE-D, subscales and all individual questions. ICC values (2,1) for the LE patients alone showed also excellent test-retest reliability for the PRTEE and subscales. Individual questions 1,2,4,8 and 9 had fair to good test-retest reliability, the other questions had excellent test-retest reliability for LE patients only. The Minimal Detectable Change for the PRTEE-D was 9.1, MDC was 5.43 for the pain subscale and 5.62 for the function subscale. Figure 1 shows the Bland and Altman plot of the PRTEE-D and Figure 2 shows this plot for LE patients only. Absolute agreement was not assumed for the PRTEE-D and pain subscale. The mean difference between test and retest for the PRTEE-D total score was 0.97 with a 95% CI of 0.11-1.83; The mean difference of the pain subscale was 0.61 with a 95% CI of 0.05-1.17. The mean difference for the PRTEE-D total score for LE patients only was 3.74 with a 95% CI of 0.49-7.00; the mean difference of the pain subscale for LE patients only was 2.58 with a 95% CI of 0.55-4.61. Absolute agreement was assumed for the function scale (mean difference 0.35, 95% CI (-0.13-0.83)), this was also the case for the LE patients only (mean difference 1.16, 95% CI -0.75-3.10).

**Table 2:** **Test-retest reliability (ICC, SEM and MDC) of the PRTEE-D, subscales and individual questions**

Pain subscale					Function subscale				
Question	ICC (95% CI) total	ICC (95% CI) LE patients	SEM total	MDC total	Question	ICC (95% CI) total	ICC (95% CI) LE patients	SEM total	MDC total
1	.87 (.81-.91)	.69 (.45-.84)	.67	1.86	6	.92 (.88-.94)	.78 (.59-.89)	.59	1.64
2	.96 (.94-.97)	.71 (.42-.86)	.57	1.59	7	.94 (.91-.96)	.82 (.66-.91)	.66	1.83
3	.94 (.92-.96)	.78 (.58-.89)	.71	1.96	8	.91 (.87-.93)	.73 (.50-.86)	.60	1.67
4	.77 (.68-.83)	.59 (.30-.78)	.59	1.64	9	.94 (.91-.96)	.74 (.53-.87)	.68	1.90
5	.99 (.98-.99)	.84 (.69-.92)	.38	1.05	10	.94 (.92-.96)	.87 (.75-.94)	.47	1.29
					11	.93 (.91-.95)	.77 (.56-.89)	.71	1.97
					12	.92 (.88-.94)	.82(.65-.91)	.50	1.37
					13	.95 (.93-.96)	.84 (.69-.92)	.54	1.49
					14	.97 (.95-.98)	.89 (.79-.88)	.45	1.25
					15	.96 (.94-.97)	.88 (.76-.94)	.52	1.45
Pain subscale	.97 (.95-.98)	.78 (.57-.90)	1.96	5.43	Function subscale	.97 (.96-.98)	.89 (.79-.95)	2.03	5.62
					PRTEE-D overall (total)	.98 (.97-.99)	.88 (.75-.94)	3.28	9.10

ICC = Intra Class Correlation, CI = Confidence Interval, SEM = Standard Error of Measurement, MDC = Minimal Detectable Change.

### Construct validity

**Figure 3:**
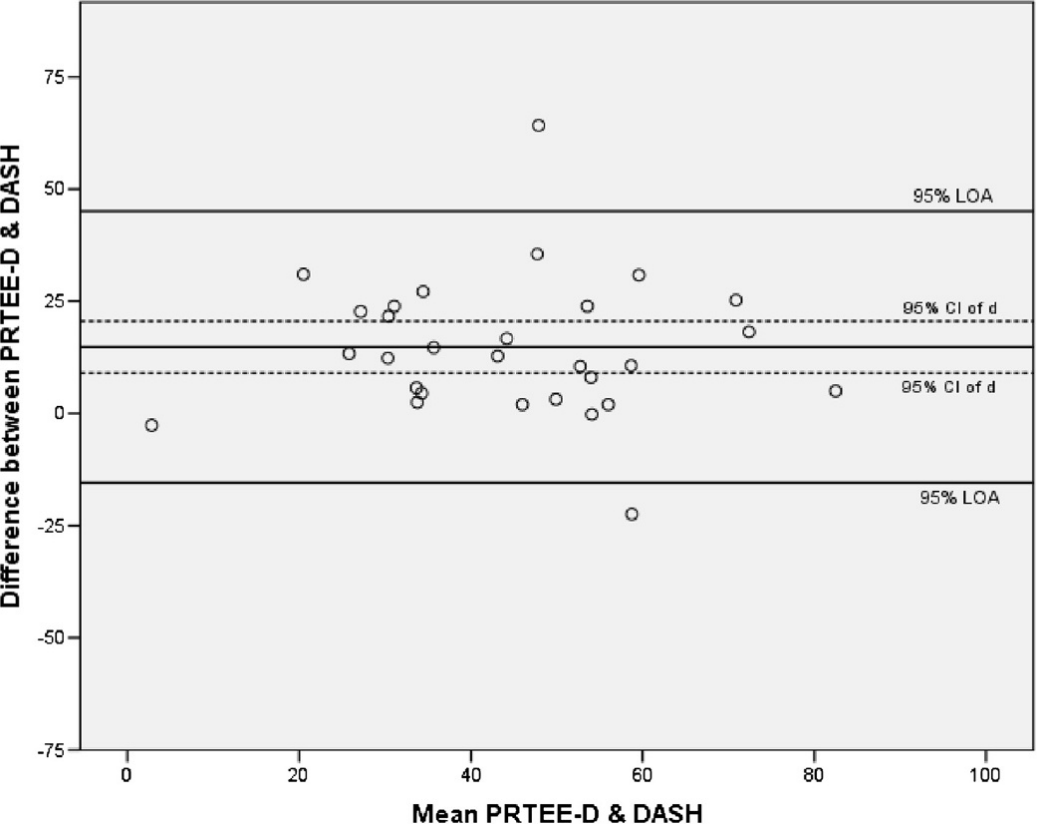
**Bland and Altman plot of construct validity PRTEE-D.**

Spearman’s correlation coefficients between PRTEE-D (subscales) and VAS pain and DASH score are provided in Table 3. The Spearman coefficients showed moderate correlations between PRTEE-D and DASH and VAS pain score. The pain subscale had a low correlation with the DASH score and a moderate correlation with VAS pain score. A moderate correlation was found between function subscale and DASH score and a high correlation was found between function subscale and VAS pain score. All correlations were significant. Figure 3 shows the Bland and Altman plot of construct validity of the PRTEE-D. The mean difference between PRTEE-D and DASH score was 14.81 with a 95% CI of 9.04-20.57. Absolute agreement was not assumed between PRTEE-D and DASH score.

**Table 3:** **Spearman’s correlation coefficients between PRTEE-D (subscales) and VAS pain and DASH score**

Measure	PRTEE-D score (p-value)	PRTEE-D pain subscale (p-value)	PRTEE-D function subscale (p-value)
DASH score	.65 (<0.01)	.45 (0.01)	.67 (<0.01)
VAS pain score	.68 (<0.01)	.55 (<0.01)	.70 (<0.01)

PRTEE-D = Patient Rated Tennis Elbow Evaluation-Dutch, DASH = Disabilities for the Arm, Shoulder and Hand questionnaire, VAS = Visual Analogue Scale.

## Discussion

Since no reliable and valid Dutch questionnaire existed to determine the severity of LE, this study aimed to translate and cross-culturally adapt the PRTEE for Dutch speaking LE patients. Semantic, idiomatic, experiential and conceptual equivalence to the original PRTEE questionnaire was assured by applying the guidelines for the process of cross-cultural adaptation of self-report measures [16].

Furthermore, reliability and validity of the PRTEE-D were determined. The total PRTEE-D score as well as the pain and function subscales demonstrated excellent internal consistency and test-retest reliability. All Spearman correlation coefficients were significant and most coefficients showed moderate correlations. We believe that these data showed good construct validity of the PRTEE-D. It can be concluded that the PRTEE-D was successfully cross-culturally adapted and is found to be a reliable and valid instrument to measure pain and functional ability in Dutch speaking patients with LE.

Cronbach’s alpha for the PRTEE-D was 0.98, this indicates an excellent internal consistency. An alpha coefficient > 0.9 is recommended for the use of a questionnaire in a clinical setting [24]. The value of Cronbach’s alpha is even slightly higher than the Cronbach’s alpha for the Italian (0.95), English (0.94), Swedish (0.94), Canadian-French (0.93) and Turkish (0.84) translations of the PRTEE [11–14, 25]. The pain (0.93) and function (0.97) subscales also showed excellent internal consistencies. These Crohnbach’s alpha values are also a little higher than the values found in other translations of the PRTEE [11–14, 25]. For LE patients only slightly lower, but still excellent values were found (0.93 total PRTEE-D, 0.80 pain subscale and 0.91 function subscale).

The ICC values show excellent values for test-retest reliability, ICC of the PRTEE-D was 0.98. The same is true for the ICC value of the pain subscale (0.97) and function subscale (0.97), all individual questions also showed excellent test-retest reliability. Test-retest reliability is in accordance with ICC values of the original questionnaire; Overend et al [8] and Newcomer et al. [9] respectively found an ICC value of 0.89 and 0.96. This also holds for the pain and function subscales, ICC values of 0.96 and 0.89 were found for the pain subscale in the original questionnaire and 0.92 and 0.83 for the function subscale [8, 9]. PRTEE questionnaires in other languages show comparable results [13, 15]. The ICC values for the LE patients only, show lower correlations, but the values of the PRTEE-D and subscales are still excellent and comparable to previous studies. A systematic bias is assumed for the PRTEE-D total score and pain subscale, because the 95% CI of the difference did not contain zero. The PRTEE-D score on the second assessment showed an almost 1 point higher score than the first measurement, LE patients showed a 3.7 point higher score on the second measurement. This might be explained by an actual improvement of symptoms due to for example treatments performed during this interval. A minor improvement is consistent with what can be expected from the literature [26]. No constraints on treatment were imposed because of ethical considerations*.* Most of the other studies which investigated test-retest reliability of the PRTEE used a shorter time interval than the one week used in this study to prevent an alteration in the severity of symptoms. A one week period was chosen in this study to prevent ‘copying’ from a subject’s memory and no longer to prevent major changes in symptoms [17].

VAS-pain and DASH questionnaire were chosen to determine construct validity of the PRTEE-D by calculating Spearman’s correlations, because an instrument that can be considered the gold standard for LE patients does not exist. The Dutch language version of the DASH questionnaire was found to be reliable and valid to assess disability and symptoms in patients with upper limb disorders [18] and VAS-pain provides an indication of pain of a LE patient. Across the different adaptation studies, several measurement tools were used to assess validity. We believe that at least the DASH should be used to assess construct validity, because this is probably the best alternative for the PRTEE, being a validated questionnaire designed to measure upper limb disabilities and symptoms [19]. The DASH was used in four of the seven PRTEE adaptation studies. Other measurement tools used to assess validity were among others the Roles and Maudsley test, VAS-pain, pain free grip and maximal grip strength. Results showed a moderate spearman correlation (0.68) between PRTEE-D (total) and VAS-pain scores. This is in resemblance with the original questionnaire (0.66) [9] and the Canadian-French PRTEE (0.77) [14]. A moderate correlation was also found between PRTEE-D and DASH score (0.65). This value is close to the correlation of the original questionnaire with the DASH (0.72). Validation studies of the PRTEE in other languages show similar correlations with the DASH questionnaire as well (Turkish 0.68, Swedish 0.88) [11, 13]. The correlation of the PRTEE pain subscale with the DASH shows the lowest correlation (0.45). The validation of the PRTEE in other languages also found the lowest correlation between the pain scale and the DASH, although the correlations were slightly higher (0.50-0.78) [11–14, 25]. The relatively low correlation between the pain scale of the PRTEE-D and DASH score is probably caused by the small number of questions on pain in the DASH questionnaire. The other correlations of the subscales with VAS and DASH show moderate to high correlations comparable to the original questionnaire and translations. A systematic bias was found between PRTEE-D and DASH score. Patients scored on average 14.8 points higher (more severe symptoms) on the PRTEE-D than on the DASH. An explanation for the relatively low correlations and systematic bias between PRTEE-D and DASH and VAS-pain score is that DASH and VAS-pain were not specifically designed for LE in contrast to the PRTEE. Excellent correlations and absolute agreement were therefore not to be expected. We can therefore state that correlations of PRTEE-D with VAS-pain and DASH show good construct validity for the PRTEE-D.

The methodology of cross-cultural adaptations of (previous versions) of the PRTEE slightly differs. All but one of the previous cross-cultural adaptations of the PRTEE used the criteria proposed by Beaton et al. [16]. This comprehensive and well described way of cross-culturally adapting questionnaires seems justified. All the studies which investigated test-retest reliability provided ICC values and all except one provided Cronbach’s alpha values [15]. One of the studies investigating the PRTEE only investigated validity [14]. To be able to use a questionnaire in clinical practice or research, sufficient reliability and validity are desirable.

A large number of participants were included in this study to investigate test-retest reliability; however a limitation of this study is that in contrast to other reliability and validity studies of the PRTEE a high number of participants without complaints was used. This might have positively influenced test-retest reliability values. However, analysis of the LE patients only showed only slightly lower values, indicating that the influence of including participants without complaints is only minimal. The LE patients included in the study seem to be a representative sample, because they were recruited in several (para)medical settings. Further, as also reported in the original and other translated versions of the PRTEE, a number of participants indicated having trouble with some questions because they never performed that activity with their injured arm. The best solution for this is probably to use the average score of the subscale for this question, like stated in the PRTEE manual for missing questions [27]. Another option, use the maximum score, seems less suitable because patients are most likely able to do the activity without unbearable pain but choose the less painful option (using their other arm). The PRTEE-D is consistent with other versions on this subject. This has to be taken into account when for example considering a LE patient with his/her non-dominant arm affected. Another limitation is that no constraints were imposed on treatments between the first and second measurement, this is however not possible due to ethical considerations. This study did not study the responsiveness of the PRTEE-D, further research still needs to address the responsiveness of this questionnaire.

## Conclusion

While Lateral Epicondylalgia is a common injury, to our knowledge no reliable and valid Dutch outcome measure specific to LE existed so far. This study showed that the PRTEE-D is successfully cross-culturally adapted and is a reliable and valid tool to measure severity of LE. It can be used as an assessment and evaluation tool, for example to monitor or determine the effects of a treatment. With the Dutch PRTEE Dutch speaking clinicians as well as researchers are now provided with a reliable, valid and easy-to-use instrument. Moreover, it is now possible to compare Dutch results from research on LE to international data [16].

## Electronic supplementary material

Additional file 1: **PRTEE-D questionnaire.** The additional file provided is the Dutch version of the PRTEE questionnaire (Patiënt- beoordeelde tennis elleboog evaluatie). (PDF 12 KB)
