# Supplementary material for: Cross-cultural adaptation and reliability and validity of the Dutch Patient-Rated Tennis Elbow Evaluation (PRTEE-D)
Source: BMC Musculoskelet Disord. 2014 Aug 11;15:270. doi: 10.1186/1471-2474-15-270 (PMC4243580; doi:10.1186/1471-2474-15-270)
Supplement: Supplementary file 1 — Additional file 1: PRTEE-D questionnaire. The additional file provided is the Dutch version of the PRTEE questionnaire (Patiënt- beoordeelde tennis elleboog evaluatie). (PDF 12 KB) [file 12891_2013_2227_MOESM1_ESM.pdf]

PATIËNT- BEOORDEELDE TENNIS ELLEBOOG EVALUATIE

Naam: ____________________________ Datum: _______________

De onderstaande vragen helpen ons om inzicht te krijgen in de mate van pijn en beperkingen die u afgelopen week aan uw arm hebt gehad. Wilt u op een schaal van 0-10 aangeven hoeveel klachten u ***gemiddeld*** *aan uw arm heeft gehad* ***gedurende de afgelopen week****. Geeft u alstublieft antwoord op alle vragen. Als u een activiteit niet heeft uitgevoerd door de pijn of omdat u niet in staat was deze activiteit te doen dient u een "10'' te omcirkelen. Als u het niet zeker weet maak dan een zo goed mogelijke schatting. Vul alleen geen antwoord in als u de betreffende activiteit nooit uitvoert. U kunt dit aangeven door de vraag in zijn geheel door te strepen.*

1. PIJN IN UW AANGEDANE ARM	
Geef uw gemiddelde mate van pijn in uw arm ***gedurende de afgelopen week*** *aan, door het cijfer te omcirkelen dat uw pijn het beste weergeeft op een schaal van 0-10. Een* ***nul*** *(0) betekent dat u* ***helemaal geen pijn heeft gehad*** *en een* ***tien*** *(10) betekent dat u* ***de ergst denkbare pijn*** *heeft gehad.*	
BEOORDEEL UW PIJN:	Geen Pijn … Ergst denkbare pijn
Wanneer u aan het rusten bent	0 1 2 3 4 5 6 7 8 9 10
Wanneer u een taak doet met herhaalde arm bewegingen	0 1 2 3 4 5 6 7 8 9 10
Wanneer u een plastic tas met boodschappen draagt	0 1 2 3 4 5 6 7 8 9 10
Wanneer uw pijn het minst was	0 1 2 3 4 5 6 7 8 9 10
Wanneer uw pijn het ergst was	0 1 2 3 4 5 6 7 8 9 10

Z.O.Z………

2. FUNCTIONELE BEPERKINGEN

A. SPECIFIEKE ACTIVITEITEN

*Geef aan **hoeveel moeite** u heeft ervaren bij het uitvoeren van onderstaande taken, gedurende de afgelopen week, door het cijfer te omcirkelen dat uw moeite het beste weergeeft op een schaal van 0-10. Een nul (0) betekent dat u helemaal geen moeite heeft ondervonden en een **tien (10)** betekent dat het **zoveel moeite kostte dat u helemaal niet in staat was het te doen.***

	Geen moeite										Niet in staat om te doen
Een deurknop of sleutel omdraaien	0	1	2	3	4	5	6	7	8	9	10
Een boodschappen tas of aktetas bij het handvat dragen	0	1	2	3	4	5	6	7	8	9	10
Een volle kop koffie of glas melk optillen en naar uw mond brengen	0	1	2	3	4	5	6	7	8	9	10
Een pot openen	0	1	2	3	4	5	6	7	8	9	10
Een broek optrekken	0	1	2	3	4	5	6	7	8	9	10
Een natte washand of handdoek uitwringen	0	1	2	3	4	5	6	7	8	9	10

B. GEBRUIKELIJKE ACTIVITEITEN

*Geef aan **hoeveel moeite** u heeft ervaren bij het uitvoeren van uw gebruikelijke activiteiten in elk van de onderstaande gebieden, gedurende de afgelopen week, door het cijfer te omcirkelen dat uw moeite het beste weergeeft op een schaal van 0-10. Met "gebruikelijke activiteiten'' bedoelen we de activiteiten die u uitvoerde **voordat** u last kreeg van uw arm. Een **nul (0)** betekent dat u helemaal **geen moeite heeft ondervonden** en een **tien (10)** betekent dat het zoveel moeite kostte dat u helemaal niet in staat was uw gebruikelijke activiteiten te doen.*

1. Persoonlijke activiteiten (aankleden, wassen)	0	1	2	3	4	5	6	7	8	9	10
2. Huishoudelijk werk (schoonmaken, onderhoud)	0	1	2	3	4	5	6	7	8	9	10
3.Werk (uw baan of dagelijks werk)	0	1	2	3	4	5	6	7	8	9	10
4. Recreatieve of sport activiteiten	0	1	2	3	4	5	6	7	8	9	10

Opmerkingen:
